# Physical Education for Girls

**Published:** 1922-10

**Authors:** 


### PHYSICAL EDUCATION FOR GIRLS.

A COMMITTEE formed in October, 1921, at the instance of the College of Preceptors, to consider the effects of physical education on girls, has drawn up its report. Representatives were appointed by the Royal College of Physicians, the Royal College of Surgeons, the British Medical Association, the Medical Women's Federation, the British Association for Physical Training, the Ling Association, the National Union of Women Teachers, the Association of Assistant Mistresses in Secondary Schools, the Private Schools Association, and the College of Preceptors. Dr. G. F. Still was Chairman of the Committee.

In reply to questionnaires 629 replies were received, of which 233 were from medical practitioners and 158 from women students. One hundred and eighty-five replies came from headmistresses. The information from medical practitioners was submitted to a special committee consisting of the medical members, who made a separate report.

Among the schoolmistresses there was a general consensus of opinion that the effects of games and physical exercise on the disposition and character of girls are beneficial; but a small number thought there was a tendency to magnify the relative importance of games, to the detriment of character. On the question whether gymnastics with apparatus is suitable for girls, although about 66 per cent. of the schoolmistresses were in favour of such gymnastics, about half of those who approved were of opinion that there was special need for careful supervision if apparatus were used. Lawn tennis and netball received general approval. Hockey, though approved by the majority of mistresses and students, was regarded by some as suitable only for the older and stronger girls and by some as too rough or strenuous a game for girls. Cricket and swimming were generally approved. Racing in rowing was condemned. Cycling, in moderation, met with a large measure of approval.

Contests in games and sports were thought to require careful limitation and grading with a view to preventing overstrain. Ill effects would be less likely to occur from the more strenuous if previous medical examination were more general.

### Medical Members' Report.

The medical members consider physical education, including games and sports, is as generally beneficial to girls as to boys. Competitive games and sports are permissible, provided that they are undertaken with due regard to the fitness of the individual. With regard to gymnastics, whether with or without apparatus, disciplined exercises under expert direction are valuable in promoting the harmonious development of the muscles and preventing faulty position and carriage. The use of apparatus entails special care, as injurious effects may come from injudicious exercise of this sort.

An important question is whether and to what degree physical exercise should be restricted during the menstrual period. Abstention from games and sports has been generally recommended hitherto, but in recent years evidence has been brought forward to show that these restrictions are harmful rather than beneficial. The medical members of the Committee are not prepared at the present time to give any final judgment on the question, but consider that the evidence justifies more extensive trial of the voluntary continuance of games, sports, and gymnastics (swimming excepted) during the menstrual period.

Perhaps the most important point which arises in connection with the physical education of girls is its influence in after-life, if any, upon motherhood. It is difficult to obtain conclusive evidence on this point. On the whole it would seem that there is no clear proof that strenuous physical education has any special influence either upon the prospect of motherhood or upon the difficulty of labour.

